# CAPE-*p*NO_2_ Inhibited the Growth and Metastasis of Triple-Negative Breast Cancer via the EGFR/STAT3/Akt/E-Cadherin Signaling Pathway

**DOI:** 10.3389/fonc.2019.00461

**Published:** 2019-06-04

**Authors:** Qin Huang, Sai Li, Liwen Zhang, Xufang Qiao, Yanyan Zhang, Xiaoyan Zhao, Guojun Xiao, Zhubo Li

**Affiliations:** College of Pharmaceutical Sciences, Southwest University, Chongqing, China

**Keywords:** EGFR, EMT, growth, metastasis, caffeic acid *p*-nitro-phenethyl ester (CAPE-*p*NO_2_), TNBC

## Abstract

Overexpressed epidermal growth factor receptor (EGFR) and overactivated epithelial-mesenchymal transition (EMT) in triple-negative breast cancer (TNBC) can enhance tumorigenesis and tumor recurrence and metastasis. Caffeic acid *p*-nitro-phenethyl ester (CAPE-*p*NO_2_) has various pharmacological activities in our previous research, but its effect on metastasis and growth of TNBC has not been studied. In this study, Caffeic acid phenethyl ester (CAPE) was as a positive control. *in vitro*, MTT, Transwell, wound healing, colony formation and cell adhesion assays were performed to examine the effect on viability, invasion, migration, colony formation and adhesion of MDA-MB-231 cells by CAPE-*p*NO_2_, the results indicated that CAPE-*p*NO_2_ significantly dose-dependently inhibited metastasis of MDA-MB-231 cells (*p* < 0.05). *in vivo*, TNBC xenograft mice were established by subcutaneously injected with MDA-MB-231 cells, and they were used to estimate the effect on metastasis and growth of CAPE-*p*NO_2_ after 38 days of treatment. HE staining and TUNEL staining were carried out in tumor tissues, results showed that CAPE-*p*NO_2_ obviously suppressed the tumor growth, induced cells apoptosis (*p* < 0.01) and decreased pulmonary and splenic metastatic tumor cells. The results of IHC demonstrated that the VEGFA and Ki-67 proteins expression were downregulated (*p* < 0.01) in tumor tissues. Furthermore, western blot analysis was used to quantify key metastasis- and growth-associated proteins expression *in vitro* and *in vivo*, the results suggested that CAPE-*p*NO_2_ downregulated the proteins expression of p-EGFR, p-STAT3, p-Akt, MMP-2, MMP-9, Survivin, and key EMT-related proteins (Vimentin and N-cadherin) (*p* < 0.01), and increased the expression of E-cadherin (*p* < 0.01) *in vivo* and *in vitro*. Besides, CAPE-*p*NO_2_ had a similar effect as erlotinib in regulating the EGFR downstream proteins in EGF-induced MDA-MB-231cells. Collectively, these results indicated that CAPE-*p*NO_2_ possessed inhibitory effect on the growth and metastasis of TNBC may via the EGFR/STAT3/Akt/E-cadherin signaling pathway, and CAPE-*p*NO_2_ is better than CAPE in inhibiting growth and metastasis.

## Introduction

Triple-negative breast cancer (TNBC) is an aggressive type of cancer with high metastasis, recurrence and a poor 5-year survival rate (1, 2). Distant metastasis remains one of the serious problems faced by TNBC patients (3, 4). Intriguingly, studies have shown that activated carcinogenic molecules are closely related to tumor progression and metastasis, and targeted depletion of these carcinogenic factors can result in decelerated tumor metastasis (5, 6). Therefore, there is a pressing need to research drugs that inhibit activated carcinogenic molecules to decrease tumors metastasis.

Epidermal growth factor receptor (EGFR), a type of transmembrane glycoprotein with PTK (protein tyrosine kinase) activity, can bind to epidermal growth factor (EGF) to form kinase-active homodimers or heterodimers. Overexpression of EGFR induces downstream PI3K/Akt and JNK/STAT intracellular signaling pathways (7, 8) that result in provoking enhancements in a plethora of biological functions, such as promotion of cell proliferation, protection against apoptosis, acceleration of invasion, and angiogenesis (9). The recognized mechanism of EGFR overexpression is that EGFR gene amplification. Approximately 25% of cases of metaplastic breast cancer have a specific phenotype of TNBC (10, 11). In addition, it has been reported that at least 50% of TNBC cases have gene amplification or high expression levels of EGFR (12, 13). Overexpression of EGFR is a key tumor promoter and has been observed in several malignancies, especially in TNBC (6, 14). Additionally, aberrant activation of EGFR has been shown to importantly participate in tumorigenesis by enhancing cell proliferation and migration and angiogenesis in aggressive TNBC (15, 16). In recent decades, anticancer medicines targeting EGFR have been intensely investigated. According to previous studies, the zinc-finger protein ZNF516 and salidroside can inhibit EGFR expression to repress the invasion and metastasis of MDA-MB-231 cells (14, 17). Therefore, TNBC patients are most likely to benefit from anti-EGFR therapies. In addition, epidermal growth factor receptor tyrosine kinase inhibitors (EGFR-TKIs) are mainly used clinically in non-small cell lung cancer patients and can significantly improve patient survival time (18). However, EGFR-TKI treatment for breast cancer has been evaluated in clinical trials, but the trials are currently underwhelming (19, 20), and there is still no available clinical drug targeting EGFR to inhibit TNBC metastasis.

Epithelial-mesenchymal transition (EMT) is a biologic process of losing epithelial cell characteristics to acquire mesenchymal cell phenotypic properties (21). The EMT process is necessary to participate in tumor development and involves an increase in invasiveness, resistance to apoptosis and extracellular matrix components (22, 23). EMT can be induced by various activation factors, including matrix metalloproteinases (MMPs), EGFR-mediated proteins (24, 25), and some miRNAs (such as miR-148a and miR-29a) (26, 27). In EMT, epithelial cells have the ability to invade the extracellular matrix and thus may contribute to tumor relapse and metastases (28). Complex genetic changes are necessary for phenotypic changes to emerge in EMT. The loss of functional E-cadherin is a common feature of EMT, and the expression level of E-cadherin and N-cadherin is a dynamic balance. EMT is partly mediated by many relative transcription factors, such as Snail and Slug, which function as direct repressors of the epithelial marker E-cadherin and inducers of the mesenchymal marker Vimentin (21, 29, 30). Interestingly, EGFR has been shown to accelerate EMT progression, and articles showed that some EGFR related proteins could regulate EMT progression in tumors (22, 25, 31).

Caffeic acid phenethyl ester (CAPE) is one of the main active components of propolis (32). CAPE can inhibit tumor cell migration and angiogenesis by efficiently blocking the EGFR signaling pathway *in vitro* and *in vivo* (33). Caffeic acid para-nitro phenethyl ester (CAPE-*p*NO_2_), a derivative of CAPE, has been designed and synthesized in our laboratory (34). It possesses higher pharmacological activities than CAPE, including anti-oxidative, anti-inflammatory, anti-colon cancer and anti-cervical carcinoma, and can protect against acute myocardial ischemia/reperfusion injury in rats and attenuate diabetic cardiomyopathy in STZ-induced diabetic mice (35–39).

In this study, the effects on the metastasis and growth of MDA-MB-231 cells by CAPE-*p*NO_2_ were investigated and compared with CAPE *in vivo* and *in vitro*. The objectives we expected to achieve are as follows: (1) to explore the inhibitory effects on the metastasis and growth of MDA-MB-231 cells by wound healing, Transwell and colony formation assays and histological anatomy of the organs of xenograft mice after treatment with CAPE-*p*NO_2_; and (2) to investigate the anti-TNBC effects and the possible mechanisms of CAPE-*p*NO_2_ on MDA-MB-231 cells and xenograft mice by analyzing the protein expression of p-EGFR, p-STAT3, p-Akt, MMP-2, VEGFA, Survivin, MMP-9, and EMT-related proteins.

## Materials and Methods

### Reagents and Ethics Statement

CAPE and CAPE-*p*NO_2_ (purity > 99.0%) were synthesized, characterized and analyzed as described in a previous study (40). High-glucose Dulbecco's modified Eagle's medium (DMEM-H), fetal bovine serum (FBS), penicillin, streptomycin, 0.25% Trypsin-EDTA, and phosphate-buffered saline (PBS) were purchased from Gibco/Invitrogen (Carlsbad, CA, USA). Dimethyl sulfoxide (DMSO), crystal violet, 3-[4,5-dimethyl-2-thi-azolyl]-2,5- diphenyl-2-tetrazolium bromide (MTT), and bovine serum albumin (BSA) were purchased from Sigma-Aldrich (St. Louis, MO, USA). Non-fat dry milk was obtained from Biofroxx (Germany). Transwell chambers and BD Matrigel™ basement membrane matrix were purchased from Corning (Corning, MA, USA). Recombinant human EGF, 4% paraformaldehyde, SDS-PAGE sample loading buffer (SDS, 5X), RIPA lysis buffer (RIPA, including 20 mM Tris, 150 mM NaCl, 1% Triton X-100, sodium pyrophosphate, β-glycerophosphate, Na_3_VO_4_, EDTA, leupeptin, etc.) and BCA protein assay kit (BCA) were obtained from Beyotime Biotechnology (Shanghai, China). Western Bright ECL (ECL) kit was obtained from Advansta (USA). Erlotinib was purchased from Selleck (USA). Primary antibodies against EGFR, p-EGFR (tyr1172), signal transducer and activator of transcription 3 (STAT3), p-STAT3 (ser727), serine/threonine kinase (Akt), p-Akt (ser473), matrix metalloproteinase-2 (MMP-2), matrix metalloproteinase-9 (MMP-9), vascular endothelial growth factor A (VEGFA), Survivin, Ki-67, E-cadherin, N-cadherin, Snail, Vimentin, GAPDH, and horseradish peroxidase (HRP)-conjugated goat anti-rabbit secondary antibody were purchased from Proteintech Group, Inc. (Wuhan, China). CAPE, CAPE-*p*NO_2_ and erlotinib were dissolved in DMSO and diluted to experimental concentrations with culture medium. This study was approved by the Ethical Committee for Animal Experiments of Southwest University (Permit Number: SYXK 2016-0002). All animal experiments were conducted in agreement with the Guide for the Care and Use of Laboratory Animals and were approved by the Committee on Animals Handling of Southwest University.

### Cell Culture

The human breast cancer cell line MDA-MB-231 was obtained from the Cell Bank at the Chinese Academy of Sciences (Shanghai, China). MDA-MB-231 cells were cultured in DMEM-H medium supplemented with 10% FBS and 1% penicillin-streptomycin solution at 37°C in a humidified 5% CO_2_ incubator.

### Cell Viability Assay and Cell Adhesion Assay

The effects of CAPE and CAPE-*p*NO_2_ on cell viability and adhesion were analyzed by an MTT assay. A total of 1 × 10^4^ cells/well were seeded into 96-well plates and incubated for 12 h and then incubated in the absence and presence of 5, 10, and 20 μM CAPE or CAPE-*p*NO_2_ for an additional 24 h. The same volume of DMSO was used as the vehicle control for CAPE and CAPE-*p*NO_2_ experiments. At the end of incubation, 10 μL MTT (5 mg/ml) were added to each well, and the cells were incubated for an additional 4 h, and the formed formazan was dissolved in 150 μL DMSO. The number of living cells was analyzed by measuring the absorbance at a wavelength of 490 nm with a microplate reader (Tecan Infinite M 1000, Austria) (38). For the cell adhesion assay, 1 × 10^4^ cells were suspended in the absence or presence of 5, 10, and 20 μM CAPE or CAPE-*p*NO_2_ and then added to the 96-well plates pre-coated with Matrigel for 2 h of incubation. The unattached cells were washed away with PBS, and the adherent cells were analyzed by MTT assay as mentioned above.

### Colony Formation Assay

Approximately 300 MDA-MB-231 cells were seeded into 35 mm dishes for 12 h and then treated with or without 5, 10, and 20 μM CAPE or CAPE-*p*NO_2_ for 2 weeks, and the drug-containing medium was renewed every 3 days. Colonies were fixed with 4% paraformaldehyde and stained with 0.05% crystal violet at room temperature. Images of the colonies were obtained using a Canon digital camera (5). All colonies were counted manually using Image-Pro Plus software (Media Cybernetics, Inc., Rockville, MD, USA).

### Wound Healing Assay

MDA-MB-231 cells (2 × 10^5^ cells/well) were seeded into 6-well plates and incubated until reaching 90% confluence. A 10 μL pipette tip was used to scratch the monolayer of cells, and the detached cells were washed twice with warm PBS and then incubated with or without 5, 10, and 20 μM CAPE or CAPE-*p*NO_2_ for 24 h. Images of the scratched regions at the time point of 0 h and after treatment with drugs for 24 h were obtained under a light microscope (Olympus U-RFLT50, Tokyo, Japan). The widths of the scratches were analyzed using Image-Pro Plus software (Media Cybernetics, Inc., Rockville, MD, USA).

### Transwell Assay

As described previously (41), MDA-MB-231 cells suspensions at a density of 1 × 10^5^ cells were seeded into a 24-well Transwell chamber with an 8-μm pore membrane in serum-free medium including 0.1% BSA in the absence or presence of 5, 10, and 20 μM CAPE or CAPE-*p*NO_2_. The lower chamber contained medium with 20% FBS. After incubation for 24 h, the cells that did not migrate located on the upper side of the membrane, were gently removed using cotton swabs, and the migrating cells on the undersurface were fixed with 4% paraformaldehyde and stained with 0.05% crystal violet. For invasion assays, Matrigel was used to pre-coat the Transwell chamber with an 8-μm pore membrane, and the following procedure was the same as the migration experiments. Images were acquired using a fluorescence microscope (Olympus, U-RFLT50, Tokyo, Japan). The numbers of invading and migrating cells were counted with Image-Pro Plus software (Media Cybernetics, Inc., Rockville, MD, USA).

### Western Blot Analysis

MDA-MB-231 cells were incubated with DMEM-H in the absence or presence of 5, 10, and 20 μM CAPE or CAPE-*p*NO_2_ for 24 h. The lysates of total cells or tumor tissues were extracted with lysis buffer with protease inhibitor. The total protein concentration was analyzed using a BCA protein assay kit according to the manufacturer's protocol. The protein samples containing 20% SDS were boiled for 10 min at 95°C and then separated on 8%-12% SDS-PAGE gels and transferred to PVDF membranes (Bio-Rad, U.S.). Then, the bolts were blocked with blocking buffer (5% non-fat dry milk in PBST) for 1.5 h. Next, the blots were incubated with primary antibodies against EGFR, p-EGFR, STAT3, p-STAT3, Akt, p-Akt, MMP-2, MMP-9, VEGFA, Survivin, Snail, E-cadherin, N-cadherin, Vimentin and GAPDH, and slowly shaken on a shaking bed overnight at 4°C. Then, the blots were incubated with the corresponding horseradish peroxidase (HRP)-conjugated secondary antibodies for 1.5 h. Finally, the bound antibodies on the blots were visualized with ECL. The protein expression levels were quantified by densitometric analysis and were first normalized to GAPDH and then normalized to the control group.

### Xenografts in Athymic Nude Mice

All animal experimental studies were approved by the Ethical Committee for Animal Experiments of Southwest University (Permit Number: SYXK 2016-0002). Four- to five-week-old female BALB/c athymic nude mice (15–17 g) were purchased from Beijing HFK Bioscience Co., Ltd. (Beijing, China), and were bred in specific pathogen-free (SPF) conditions. When the nude mice adapted to the environment for 1 week, 5 × 10^6^ MDA-MB-231 cells in the exponential growth phase were re-suspended in 200 μL PBS and injected into each mouse subcutaneously. Treatment began when the tumor volume reached approximately 200 mm^3^ at 2 weeks after tumor cell injection. Xenograft mice were administered drugs by intraperitoneal injection. Thirty xenograft mice were randomly divided into the five groups as follows: (1) the xenograft mouse group was treated with 2% DMSO; (2) the CAPE group was treated with CAPE at a dose of 10 mg/kg/day; and (3) the CAPE-*p*NO_2_ groups (groups 3, 4, and 5) were treated with CAPE-*p*NO_2_ at doses of 5, 10, and 20 mg/kg/day, respectively. Subsequently, tumor volumes were analyzed every 2 days using an electronic caliper and were quantitated according to the following formula: 0.52 × length × (width)^2^ (36). After treatment for 38 days, the mice were euthanized, and solid tumors, lungs and spleens were collected, and all samples were analyzed.

### Histology and Immunohistochemistry

Excised tumors and organs were washed with ice-cold physiological salt solution and then fixed with 4% paraformaldehyde at 4°C overnight. Then, these tissues were embedded in paraffin and cut into 5 μm slices. Deparaffinized and rehydrated slices were stained with hematoxylin-eosin (HE). Then, the stained sections were observed under a microscope (Olympus, U-RFLT50, Tokyo, Japan).

For immunohistochemical (IHC) analysis, tumor tissue sections (5 μm) were incubated with primary antibodies (VEGFA and Ki-67) at 4°C for 12 h and subsequently incubated with anti-mouse HRP secondary antibody for 1 h at room temperature. The sections were rinsed in PBS, used diaminobenzidine to coloration, followed by counterstaining with hematoxylin (38). Images were acquired using a microscope (Olympus U-RFLT50, Tokyo, Japan). The images were analyzed using Image-Pro Plus software (Media Cybernetics, Inc., Rockville, MD, USA).

For TUNEL staining, the endogenous peroxidase activity was blocked with 3% H_2_O_2_ and the tumor tissue sections were incubated with terminal deoxynucleotidyl transferase (TdT) reaction buffer along with TdT reaction mixture. Then, the sections were incubated with a fluorophore-conjugated secondary antibody. The sections were stained with DAPI. Images were obtained using a fluorescence microscope (Olympus, U-RFLT50, Tokyo, Japan). The fluorescence intensity of images was analyzed using Image-Pro Plus software (Media Cybernetics, Inc., Rockville, MD, USA) (42).

### Statistical Analysis

All experiments were performed at least in triplicate. Data were analyzed by SPSS 16.0 software (SPSS, Inc., Chicago, IL, USA) and are presented as the mean value ± SD. *P* < 0.05 was considered statistically significant.

## Results

### Effects on the Viability and Colony Formation

**Figure 1:**
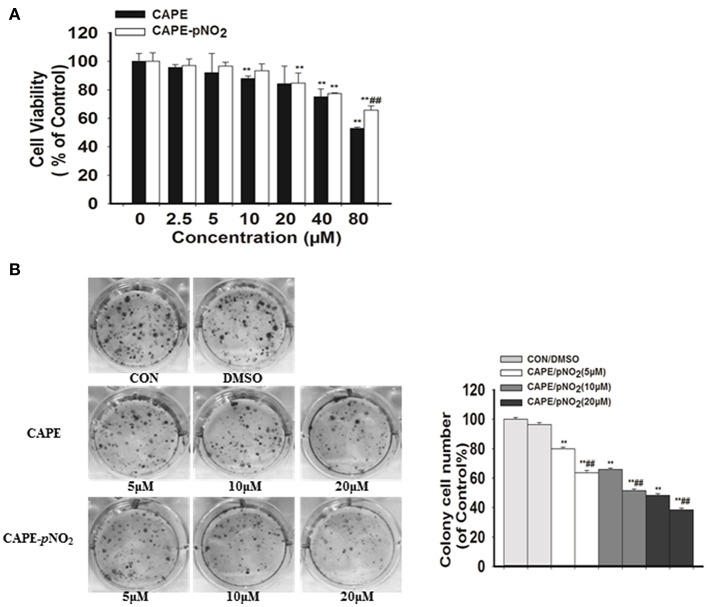
The effect of CAPE-*p*NO_2_ on inhibiting the viability and colony formation ability of MDA-MB-231 cells. **(A)** Cell viability was analyzed by an MTT assay, and cells were incubated with CAPE or CAPE-*p*NO_2_ (2.5–80 μM) for 24 h. **(B)** Colony formation ability was analyzed by a colony formation assay, and cells were incubated with CAPE or CAPE-*p*NO_2_ (5, 10, and 20 μM) for 24 h. Values represent the mean ± SD from three independent experiments; *p <0.05, **p<0.01: DMSO, CAPE and CAPE-*p*NO_2_ compared with the control; ^#^*p*<0.05, ^##^*p*<0.01: CAPE-*p*NO_2_ compared with CAPE at the same concentration.

The MTT assay showed that low dosages (2.5, 5, and 10 μM) of CAPE-*p*NO_2_ exhibited no significant effect on the viability of MDA-MB-231 cell line, while cell viability was dose-dependently inhibited at higher doses (20, 40, and 80 μM) of CAPE or CAPE-*p*NO_2_ (Figure 1A). Clones were larger and more prevalent in the control, and the opposite results were observed in the drug-treated groups (Figure 1B). The results indicated that the colony formation ability of cells was significantly inhibited by CAPE and CAPE-*p*NO_2_, and CAPE-*p*NO_2_ had a more pronounced effect compared with CAPE (*p* < 0.01).

### Effect on the Invasion, Migration and Adhesion

**Figure 2:**
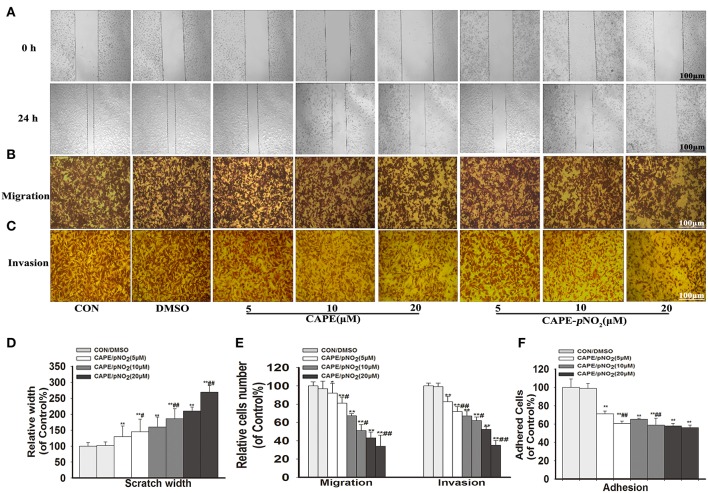
CAPE-*p*NO_2_ inhibited cell migration, invasion and adhesion abilities *in vitro*. MDA-MB-231 cells were incubated with CAPE or CAPE-*p*NO_2_ (5, 10, and 20 μM) for 24 h. **(A)** Migration ability was analyzed using a wound healing assay. **(B,C)** The cell migration and invasion abilities were analyzed using a Transwell assay. **(D,E)** A graphical representation of quantitative data showing the relative width of wound healing and the number of migrated and invaded cells. The data were analyzed by Image-Pro Plus software. **(F)** The cell adhesion ability was analyzed by an adhesion assay, and cells were incubated with CAPE or CAPE-*p*NO_2_ (5, 10, and 20 μM) for 2 h. Values represent the mean ± SD from three independent experiments; *p < 0.05, **p < 0.01: DMSO, CAPE and CAPE-*p*NO_2_ compared with the control; ^#^*p*<0.05, ^##^*p*<0.01: CAPE-*p*NO_2_ compared with CAPE at the same concentration.

The results showed that width of the wound of different concentrations groups (5, 10, and 20 μM) of CAPE or CAPE-*p*NO_2_ were narrower than the control (Figure 2A), and their cells on the lower layer of the Transwell chamber were lesser than the control (Figures 2B,C). It indicated that above two compounds could significantly inhibit the migration and invasion of MDA-MB-231 cells (Figures 2D,E) (*p* < 0.01). Besides, the number of adhered cells of the treated groups were also reduced (Figure 2F) (*p* < 0.01). Particularly, CAPE-*p*NO_2_ had evidently stronger effect against wound healing, migration and invasion at the same dosages compared with CAPE (*p* < 0.05 or *p* < 0.01).

### Effect on the Protein Expression of the EGFR Signaling Pathway

**Figure 3:**
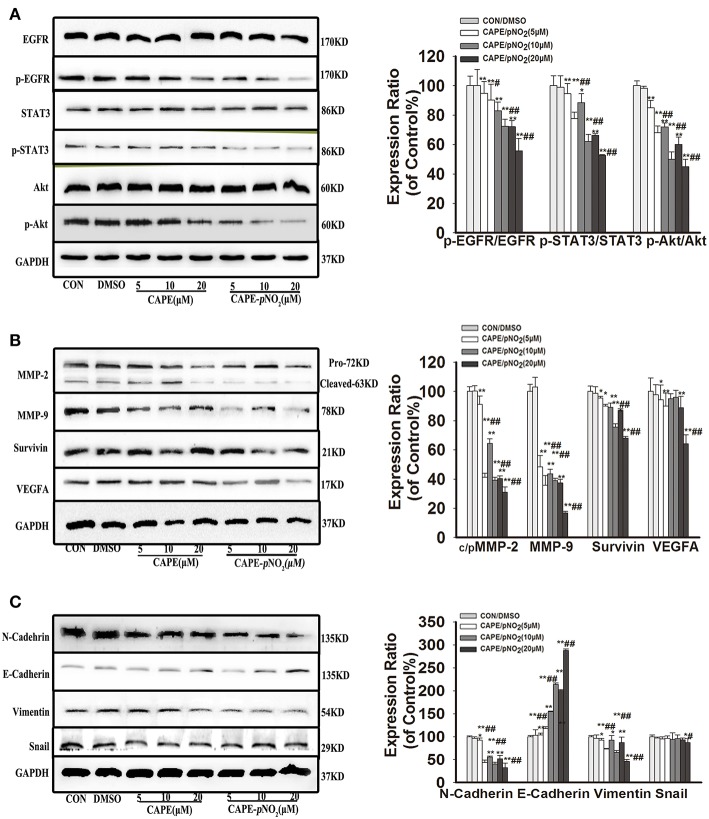
The EGFR signaling pathway and EMT progression were suppressed by CAPE-*p*NO_2_ in MDA-MB-231 cells. Cells were incubated with CAPE or CAPE-*p*NO_2_ (5, 10, and 20 μM) for 24 h, and protein expression levels were analyzed by a Western blot assay and quantified by densitometric analysis. **(A)** The protein expression levels of EGFR, p-EGFR, STAT3, p-STAT3, Akt, and p-Akt. **(B)** The protein expression levels of MMP-2, MMP-9, Survivin, and VEGFA. **(C)** EMT-related protein expression. The bar graphs show the relative protein expression levels. Values represent the mean ± SD from three independent experiments; *p < 0.05, **p < 0.01: DMSO, CAPE and CAPE-*p*NO_2_ compared with the control; ^#^*p* < 0.05, ^##^*p* < 0.01: CAPE-*p*NO_2_ compared with CAPE at the same concentration.

As shown in Figure 3, the results displayed that CAPE and CAPE-*p*NO_2_ downregulated the protein expression of p-EGFR, p-STAT3, and p-Akt (*p* < 0.01), but their total proteins were unaffected (Figure 3A). The expression levels of the metastasis-associated proteins (MMP-2 and MMP-9) and EMT-associated proteins (N-cadherin and Vimentin) were also significantly reduced (Figure 3B, *p* < 0.01), there is no clear effect on Snail. The downregulation of Survivin was also observed, and VEGFA expression was decreased only at the concentration of 5 and 20 μM by CAPE-*p*NO_2_ (Figures 3B,C). Conversely, the expression of E-cadherin, an epithelial biomarker, was upregulated (Figure 3C, *p* < 0.01). It indicated that the EGFR/STAT3/Akt signaling pathway and EMT progression were restrained by CAPE and CAPE-*p*NO_2_.

### Effect of CAPE-*p*NO_2_ on the EGF-Induced EGFR Signaling Pathway

**Figure 4:**
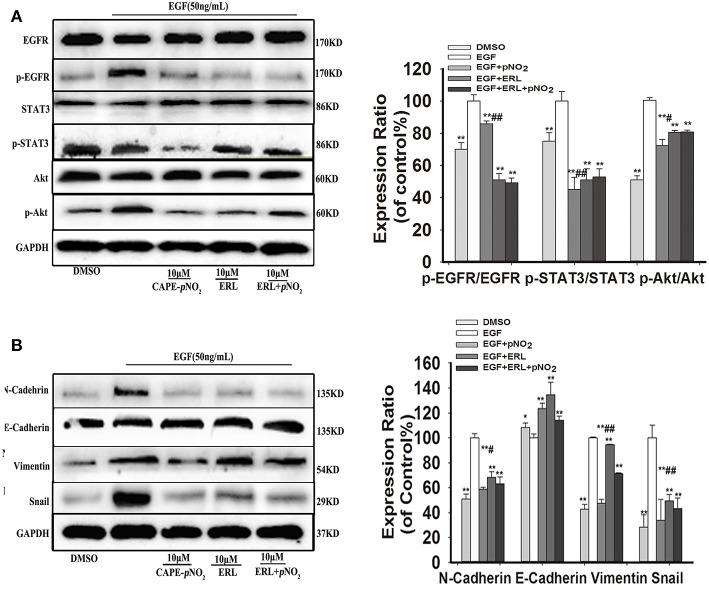
CAPE-*p*NO_2_ inhibited the EGF-induced EGFR signaling pathway. Cells were stimulated with 50 ng/ml EGF for 30 min, then replacement of culture medium containing 10 μM erlotinib (ERL) for 12 h, finally replacement of culture medium containing 10 μM CAPE-*p*NO_2_ for 24 h. Western blot analysis was performed with related antibodies and quantified by densitometric analysis. **(A)** The protein expression levels of EGFR, p-EGFR, STAT3, p-STAT3, Akt, and p-Akt. **(B)** The protein expression levels of EMT-related proteins. The bar graphs show the relative protein expression levels. Values represent the mean ± SD from three independent experiments; *p < 0.05, **p < 0.01: the treated groups compared to the EGF group; ^#^*p* < 0.05, ^##^*p* < 0.01: the CAPE-*p*NO_2_ group compared to the ERL+ CAPE-*p*NO_2_ group.

Cells were stimulated with 50 ng/mL EGF for 30 min, then replacement of culture medium containing 10 μM erlotinib for 12 h, finally replacement of culture medium containing 10 μM CAPE-*p*NO_2_ for 24 h. EGF activated the expression of p-EGFR, p-STAT3, p-Akt, and regulated EMT-associated proteins (N-cadherin, Snail, Vimentin and E-cadherin), CAPE-*p*NO_2_ and erlotinib repressed their activation, but regulations of above proteins were not significantly reinforced in drugs combination group (ERL + CAPE-*p*NO_2_) (Figures 4A,B). It suggested that CAPE-*p*NO_2_ had the similar effects as erlotinib in regulating the EGFR downstream proteins, and EMT related proteins could be control by EGFR/STAT3/Akt signal pathway in EGF-induced MDA-MB-231 cells.

### Effect on Xenograft Tumor Growth

**Figure 5:**
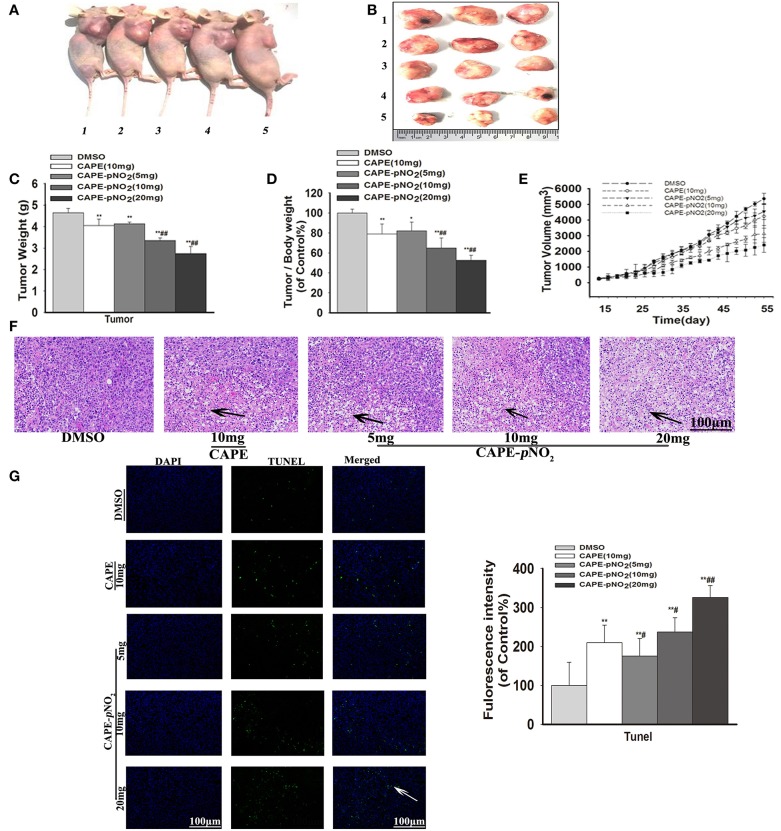
Antitumor activity of CAPE-*p*NO_2_ in xenograft tumors. Xenograft mice were treated with either DMSO (2%) (vehicle group), CAPE (10 mg/kg/day) or CAPE-*p*NO_2_ (5, 10, and 20 mg/kg/day) for 38 days. **(A)** Images of euthanized mice. Groups 1, 2, 3, 4, and 5 represented the DMSO (2%) group, the CAPE (10 mg/kg/day) group and the CAPE-*p*NO_2_ groups (5, 10, and 20 mg/kg/day), respectively. **(B)** Images of excised tumors. **(C)** Xenograft breast tumor weights. **(D)** Tumor/body weights. **(E)** The progression of xenograft breast tumor volume from different experimental groups. **(F)** HE staining of tumor sections (Black arrows indicate the necrotic area). **(G)** TUNEL staining of tumors (White arrows points to apoptotic cancer cells). The bar graph shows the fluorescence intensity statistics analyzed by Image-Pro Plus software. Values represent the mean ± SD from three independent experiments; *p < 0.05, **p < 0.01: CAPE and CAPE-*p*NO_2_ compared with DMSO group; ^#^*p* < 0.05, ^##^*p* < 0.01: CAPE-*p*NO_2_ (5, 10 and 20 mg/kg) compared with CAPE (10 mg/kg).

The tumor growth in xenograft mice was limited in the drug treatment groups. As shown in Figures 5A,B, obvious smaller xenograft tumors were observed in the treated groups compared with the DMSO group at the end of the experiment. The weight of the xenograft tumors was significantly lighter compared with the DMSO group at finished treatment (Figure 5C) (*p* < 0.01). The tumor-to-body weight ratio was lower in treated groups (Figure 5D) (*p* < 0.01). Furthermore, the cure of Figure 5E displayed that xenograft tumor volumes in treated groups were still smaller than DMSO group during administration times. In contrast, the tumors in the control were more aggressive. Particularly, CAPE-*p*NO_2_ had stronger effects on inhibiting growth than CAPE at the same dose (*p* < 0.01).

### Effect on the Apoptosis of Tumor Cells

As observed in HE staining, the necrotic areas showed relatively shallow colors (Black arrows) of tumor slices (36). The necrotic areas expanded after treatment with the compounds, as shown in Figure 5F. TUNEL staining of tumor slices showed that the number of apoptotic tumor cells (White arrow) were increased (Figure 5G, *p* < 0.01) (42). The results indicated that apoptosis of tumor cells was induced by CAPE and CAPE-*p*NO_2_.

### Effect on the Protein Expression of the EGFR Signaling Pathway *in vivo*

**Figure 6:**
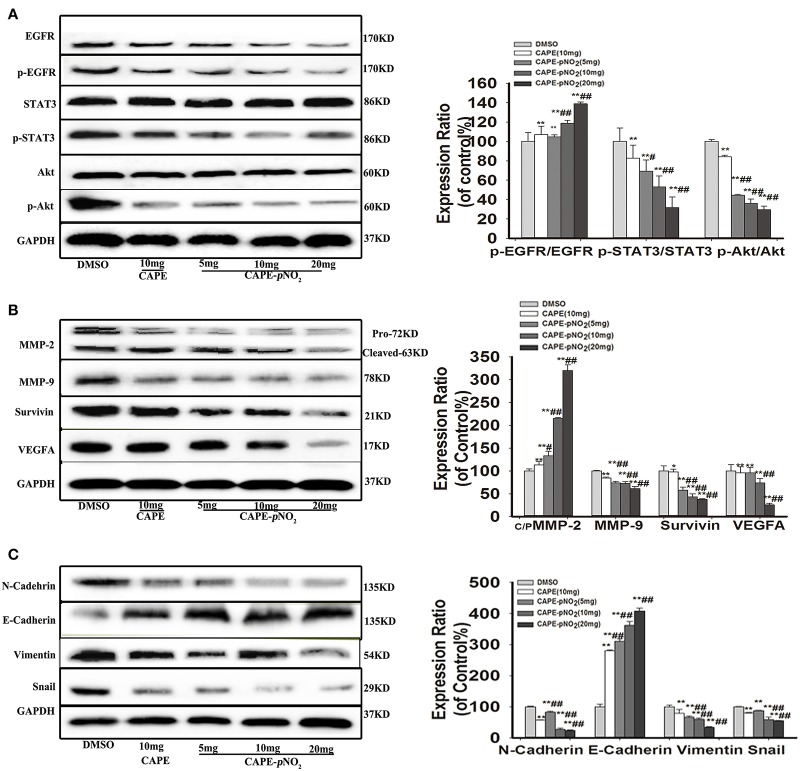
The EGFR/STAT3/Akt signaling pathway and EMT progression were suppressed by CAPE-*p*NO_2_ in tumors. The xenograft mice were treated with CAPE (10 mg/kg/day) and CAPE-*p*NO_2_ (5, 10, and 20 mg/kg/day) for 38 days. Western blot analysis was performed with related antibodies and quantified by densitometric analysis. **(A)** The protein expression levels of EGFR, p-EGFR, STAT3, p-STAT3, Akt, and p-Akt. **(B)** The protein expression levels of MMP-2, MMP-9, Survivin, and VEGFA. **(C)** EMT-related protein expression. The bar graphs show the relative protein expression levels. Values represent the mean ± SD from three independent experiments; *p < 0.05, **p < 0.01: CAPE and CAPE-*p*NO_2_ compared with DMSO group; ^#^*p* < 0.05, ^##^*p* < 0.01: CAPE-*p*NO_2_ (5, 10 and 20 mg/kg) compared with CAPE (10 mg/kg).

As shown in Figure 6A, the protein expression levels of p-EGFR, p-STAT3 and p-Akt were markedly lower in the treated groups (*p* < 0.01). There were no significant changes in the expression of STAT3 and Akt, but EGFR expression was downregulated (*p* < 0.01). The expression levels of metastasis-associated proteins (MMP-2, MMP-9, and VEGFA), Survivin and EMT-associated proteins (N-cadherin, Snail, and Vimentin) were also significantly reduced (Figure 6B, *p* < 0.01). Conversely, the expression of E-cadherin, an epithelial biomarker, was upregulated (Figure 6C, *p* < 0.01).

### Effect on VEGFA and Ki-67 Were Observed by Immunohistochemistry

**Figure 7:**
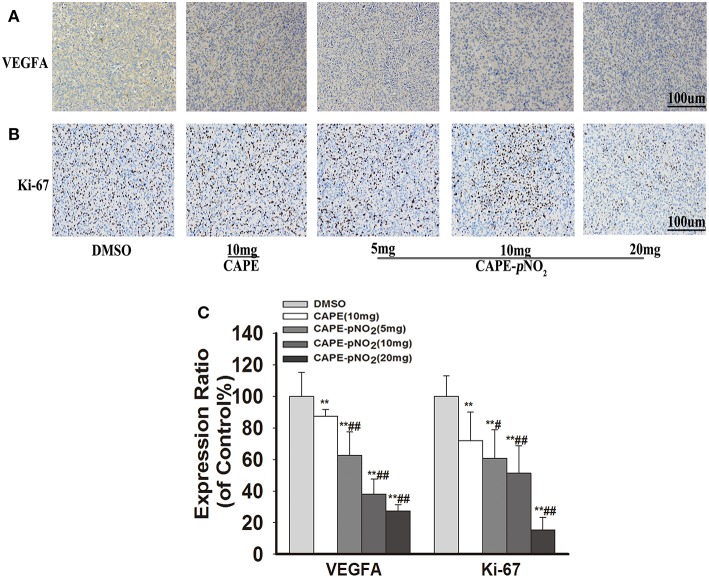
CAPE-*p*NO_2_ reduced the protein expression levels of VEFGA and Ki-67 in tumor tissues. As indicated by IHC, the brown areas indicated the positive proteins expression of VEGFA and Ki-67. The integrated option density value was used to analyze the protein expression level. **(A)** The protein expression of VEGFA. **(B)** The protein expression of Ki-67. **(C)** A graphical representation of quantitative data shows the relative proteins expression of VEGFA and ki-67. Values represent the mean ± SD from three independent experiments; *p < 0.05, **p < 0.01: CAPE and CAPE-*p*NO_2_ compared with DMSO group; ^#^*p* < 0.05, ^##^*p* < 0.01: CAPE-*p*NO_2_ (5, 10 and 20 mg/kg) compared with CAPE (10 mg/kg).

For immunohistochemistry staining, the brown areas indicate positive proteins expressions of VEGFA and Ki-67 in tumor slices (43). Figures 7A,B, the staining pictures of VEGFA and Ki-67, respectively. The expression of VEGFA and Ki-67 was decreased after treatment with CAPE or CAPE-*p*NO_2_ (Figure 7C, *p* < 0.01), and CAPE-*p*NO_2_ performed slightly better (*p* < 0.01, *p* < 0.05).

### CAPE-*p*NO_2_ Decreased Pulmonary and Splenic Metastatic Tumor Cells

**Figure 8:**
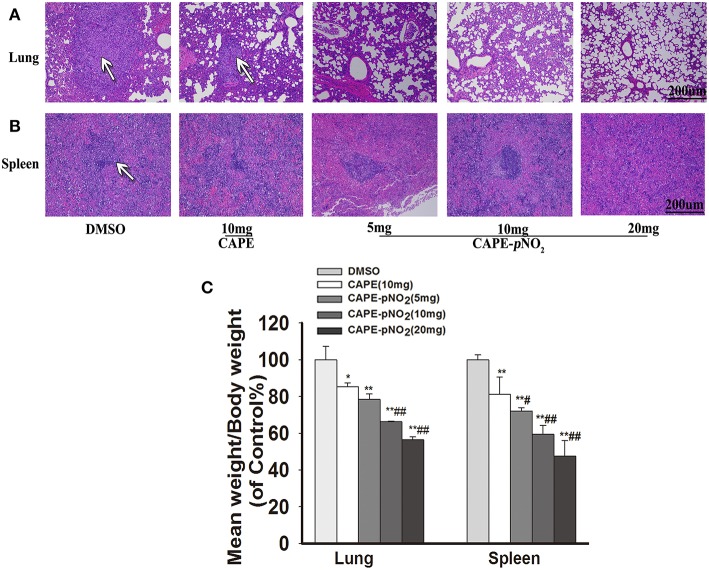
CAPE-*p*NO_2_ attenuated pulmonary and splenic metastatic tumor cells. **(A,B)** the tumor metastatic nodules (White arrows) in organs analyzed by HE staining. **(C)** The weights of the removed lungs and spleens of xenograft mice after treatment (*n* = 6). Values represent the mean ± SD from three independent experiments; *p < 0.05, **p < 0.01: CAPE and CAPE-*p*NO_2_ compared with DMSO group; ^#^*p* < 0.05, ^##^*p* < 0.01: CAPE-*p*NO_2_ (5, 10 and 20 mg/kg) compared with CAPE (10 mg/kg).

In xenograft mice, the effects of metastasis on lung and spleen tissues were observed by HE staining (44). As shown in Figure 8A, in DMSO group, the tumor metastatic nodules in the lung were larger (White arrows), the normal lung structures were almost occupied by metastatic tumor cells, and there were almost no normal small vacuoles in the lung. However, the metastasis was decreased in drugs treatment groups. Excitingly, in CAPE-*p*NO_2_ (20 mg/kg) group, only small tumor metastatic nodules were observed, and the normal structures of lung were not damaged significantly. For spleen (Figure 8B), spleen structure was infiltrated by many tumor cells, but the metastatic tumor nodules of the spleen were not as obvious as that of the lung. The weights of the dissected lungs and spleens were distinctly lower in drugs treated groups (Figure 8C, *p* < 0.01).

## Discussion

Overexpressed EGFR and overactivated EMT in TNBC can enhance tumorigenesis and tumor recurrence and metastasis, and metastasis is currently a limitation of TNBC treatment. There is a lack of an approved targeted drug therapy for TNBC against its metastasis (45).

Abundant evidence has indicated that the progression and metastasis of TNBC is closely correlated with the degree of abnormal activation of the expression of proteins, such as EGFR, Survivin, and EMT-associated proteins, etc. (6, 12, 46). It has been reported that EGFR is overexpressed in over 50% of TNBC patients and has a vital role in cell proliferation and migration, angiogenesis and protection against apoptosis (9, 12, 13), and the activation of EGFR mediates downstream signal transduction, including PI3K/Akt and JNK/STAT (7, 8).

In the present study, the effects of CAPE-*p*NO_2_ on metastasis were explored in MDA-MB-231 cells and compared with CAPE *in vivo* and *in vitro*. The cell activity rate was >80% cultivated for 24 h at 20 μM CAPE-*p*NO_2_, and no noticeable organ toxicity was observed in xenograft mice, it was consistent with recent empirical evidence regarding cancers (36, 37). The results of the Transwell, wound healing and cell adhesion assays showed that CAPE-*p*NO_2_ markedly suppressed the invasion, migration and adhesion of MDA-MB-231 cells (Figure 2). These results indicated that CAPE-*p*NO_2_ effectively suppressed the metastasis of TNBC. In xenograft mice, CAPE-*p*NO_2_ remarkably inhibited the growth of the xenograft tumor (the tumor inhibition rate was 57% at high doses), increased apoptosis of tumor cells, and decreased the mass indexes of the lung and spleen after treatment for 38 days. Enlarged lung and splenomegaly were clearly observed in tumor-bearing mice, and numerous metastatic foci were observed in the lung and spleen, indicating that CAPE-*p*NO_2_ can effectively prevent and control the prevalent phenomenon. The effect of resisting metastatic tumor to the lung has been observed in other study (47).

CAPE-*p*NO_2_ decreased the protein expression levels of p-EGFR, p-STAT3 and p-Akt *in vivo* and *in vitro*. Aberrant expression of EGFR has been observed in multiple malignancies, and activated molecules can irritate the PI3K/Akt and JNK/STAT signaling pathways, which contribute to tumor metastasis (7, 8). The EGFR expression level in MDA-MB-231 cells was relatively higher compared with other breast cancer cells (14). This result was consistent with the results reported by Verma et al. (48), further explaining the inhibitory effect of CAPE-*p*NO_2_ on breast cancer metastasis by regulating the EGFR/STAT3/Akt signal pathway. In addition, the EGFR expression level was notably reduced by CAPE-*p*NO_2_ *in vivo* only. In this regard, this result was likely because of the difference in the duration of drug action (treatment for 24 h in cells, but for 38 days in xenograft mice).

The expression levels of MMP-2, MMP-9 were dramatically declined after treatment with CAPE-*p*NO_2_ *in vivo* and *in vitro*. MMP-2 and MMP-9 are members of the matrix metalloproteinase (MMP) family, which play an important role in angiogenesis, wound healing and tumor infiltration. Tumor cells secrete VEGFA and metalloproteinases, which facilitate angiogenesis and digest the extracellular matrix to provide conditions for tumor migration and invasion. Vascular endothelial growth factor A (VEGFA) is also involved in angiogenesis and metastasis. Many studies have sufficiently confirmed that restraining the expression of MMP-2, MMP-9, and VEGFA can inhibit tumor growth, metastasis and angiogenesis (17, 27, 49). Therefore, CAPE-*p*NO_2_ against the growth and metastasis of breast cancer might be via restraining MMP-2, MMP-9, and VEGFA expression, and these proteins were associated with the EGFR/STAT3/Akt signaling pathway.

The proteins expression of Survivin and Ki-67 were inhibited by CAPE-*p*NO_2_. Survivin is an anti-apoptosis protein, which highly expressed in most human cancers and closely correlated to tumor cell proliferation, poor diagnosis and therapeutic resistance. It has been reported that reducing the expression level of Survivin can repress tumor growth and promote apoptosis (6, 46). Ki-67 is located in the nucleus, overexpressed Ki-67 has been shown to be significantly associated with proliferation of TNBC (50). In the present experiment, CAPE-*p*NO_2_ decreased the growth of breast cancer by remarkably downregulated Survivin and Ki-67 expression *in vivo*.

CAPE-*p*NO_2_ significantly reduced the expression levels of EMT-associated proteins *in vivo* and *in vitro*, such as N-cadherin, Snail and Vimentin. EMT is a reversible biological process, and it can be reversed to mesenchymal-epithelial transition (MET) by upregulating E-cadherin expression. EMT plays a vital role in developing tumors, including invasiveness, fibroid cell morphology, and EMT is closely related to drug resistance. (22, 51). Importantly, EMT potentiates cell motility properties, which can be activated by p-EGFR, MMP-2, MMP-9 and VEGFA (24, 25, 52). Studies have shown that it is feasible to inhibit tumor growth and metastasis by regulating these EMT-associated proteins (23). Prominently, CAPE-*p*NO_2_ increased the expression level of E-cadherin that reduced EMT progress in our results. The regulating effects on the EMT-associated protein of CAPE-*p*NO_2_ can weaken the invasion of MDA-MB-231 breast cancer cells.

CAPE-*p*NO_2_ depressed the upregulation of p-EGFR, p-STAT3, p-Akt, and EMT-associated protein expression, excluding E-cadherin in EGF-induced MDA-MB-231 cells, and CAPE-*p*NO_2_ had similar effects as erlotinib, an EGFR-TKI (53). Kang, D. has speculated that salidroside exerts anti-cancer effect by combing with EGFR site (17). Possibly, CAPE-*p*NO_2_ could be considered an inhibitor of EGFR phosphorylation. However, more sufficient evidence is needed for further research.

**Figure 9:**
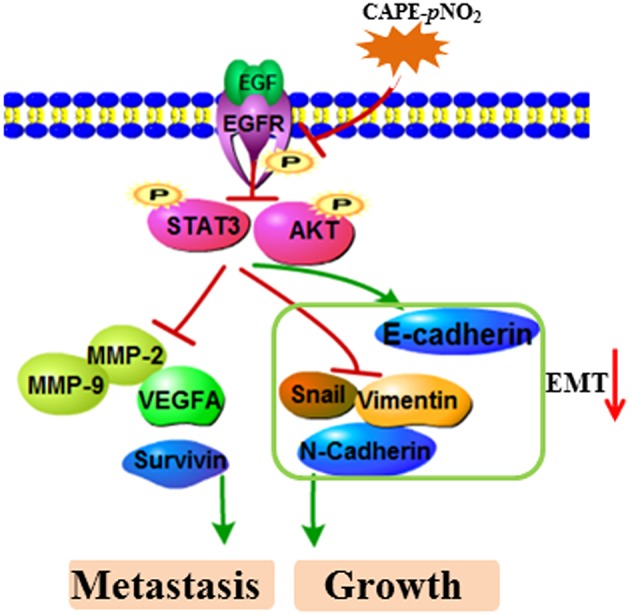
The possible mechanism of CAPE-*p*NO_2_ inhibits TNBC growth and metastasis. CAPE-*p*NO_2_ suppressed the expression of metastasis- and growth-associated proteins by restraining the EGFR/STAT3/Akt signal pathway, and EMT could be regulated by the signal pathway.

In the present study, these results demonstrated a relatively stronger effect of CAPE-*p*NO_2_ compared to CAPE on inhibiting the growth and metastasis of breast cancer. In previous studies, CAPE-*p*NO_2_ was shown to possess some higher pharmacological activities, including antioxidative, anti-inflammatory, anti-colon cancer, and anti-cervical carcinoma activities compared with CAPE (35–37). First, the nitro group is an electron-withdrawing group, which was introduced into CAPE to improve its polarity and activities (54, 55). Second, growing evidence has disclosed that the EMT process positively adjusts chemoresistance (51). In this study, CAPE-*p*NO_2_ had more pronounced effects on regulating EMT-associated protein expression *in vivo* and *in vitro*, thus, tumor cells showed higher sensitivity to CAPE-*p*NO_2_. Generally, the experimental results evidenced that CAPE-*p*NO_2_ likely via inhibiting the EGFR/STAT3/Akt/E-cadherin signaling pathway to against growth and metastasis of breast cancer *in vivo* and *in vitro*. The possible mechanism of CAPE-pNO2 inhibits TNBC growth and metastasis was summarized of Figure 9.

## Ethics Statement

This study was approved by the Ethical Committee for Animal Experiments of Southwest University (Permit Number: SYXK 2016-0002). All animal experiments were conducted in agreement with the Guide for the Care and Use of Laboratory Animals and were approved by the Committee on Animals Handling of Southwest University.

## Author Contributions

ZL and QH designed the project. QH and SL performed the mice and cell experiments. ZL, QH, SL, LZ, XQ, XZ, YZ, and GX wrote the main manuscripts. QH analyzed and interpreted data. All authors reviewed the manuscript.

### Conflict of Interest Statement

The authors declare that the research was conducted in the absence of any commercial or financial relationships that could be construed as a potential conflict of interest. The reviewer JY declared a shared affiliation, though no other collaboration, with one of the authors (ZL) to the handling Editor.

## Abbreviations

EGFR: Epidermal growth factor receptor
EMT: Epithelial-mesenchymal transition
MET: Mesenchymal-epithelial transition
TNBC: Triple-negative breast cancer
CAPE-*p*NO_2_: Caffeic acid *p*-nitro-phenethyl ester
CAPE: Caffeic acid phenethyl ester
FBS: Fetal bovine serum
DMSO: Dimethyl sulfoxide
DAPI, 4': 6-diamidino-2-phenylindole
PBS: Phosphate-buffered saline
BCA: BCA protein assay kit
ECL: Western Bright ECL
MTT, 3-[4, 5-dimethyl-2-thi-azolyl]-2: 5- diphenyl-2-tetrazolium bromide
EGFR-TKIs: Eepidermal growth factor receptor tyrosine kinase inhibitors
STAT3: Signal transducer and activator of transcription 3
Akt: Serine/threonine kinase
MMP-2: Matrix metalloproteinase-2
MMP-9: Matrix metalloproteinase-9
VEGFA: Vascular endothelial growth factor A
TUNEL: Terminal dexynucleotidyl transferase (TdT)-mediated DUTP nick end labeling.
